# Hypomethylation of a CpG Site in the CpG Island of the Aquaporin 1 Gene May Be Involved in the Formation of Adult-Onset Non-communicating Hydrocele Testis

**DOI:** 10.7759/cureus.47651

**Published:** 2023-10-25

**Authors:** Mami Hattori-Kato, Yumiko Okuno, Masayoshi Zaitsu, Hiroshi Fukuhara, Akira Nomiya, Koji Mikami, Takumi Takeuchi

**Affiliations:** 1 Department of Urology, Japan Organization of Occupational Health and Safety, Kanto Rosai Hospital, Kawasaki, JPN; 2 Center for Research of the Aging Workforce, University of Occupational and Environmental Health, Kitakyushu, JPN; 3 Department of Urology, Kyorin University Faculty of Medicine, Tokyo, JPN

**Keywords:** single-nucleotide polymorphism, water channel, methylation, hydrocele testis, aquaporin 1

## Abstract

Background

Water channel aquaporin 1 (AQP1) protein expression is enhanced in the tunica vaginalis of patients with adult-onset non-communicating hydrocele testis and may contribute to the development of non-communicating hydrocele testis. We performed genetic and epigenetic analyses of the *AQP1 *gene in the tunica vaginalis of patients with adult-onset non-communicating hydrocele testis to elucidate the cause of enhanced AQP1 protein expression.

Methodology

The genotype was determined for Tag single-nucleotide polymorphisms (SNPs) representing the *AQP1* gene and SNPs in the 5’-upstream region of the *AQP1* gene. Then, by performing association analysis, the applicability of various genetic models was investigated for each SNP. Moreover, the methylation rate of CpG sites was examined for the CpG island related to the *AQP1* gene.

Results

There was no significant association between each SNP and hydrocele testis for any of the genetic models. The average methylation rate of the 17 CpG sites evaluated was not significantly different between controls and hydrocele testis, but the methylation rate was lower in hydrocele testis than in controls at one CpG site.

Conclusions

There was a significant decrease in the methylation rate at one of the CpG sites in the CpG island associated with the *AQP1* gene in the tunica vaginalis of patients with non-communicating hydrocele testis. This may increase AQP1 protein expression and contribute to the formation of hydrocele testis. SNPs related to the *AQP1* gene were not associated with hydrocele testis.

## Introduction

Hydrocele testis involves the accumulation of clear fluid between the tunica vaginalis and testis. Adult-onset, primary, non-communicating hydrocele testis causes progressive swelling and local discomfort on the scrotum, which has been attributed to the enhanced influx and/or impaired absorption of fluid in the space between the tunica vaginalis and testis.

Aquaporins (AQPs) are a family of water channels that are conserved from lower organisms to mammals. Thirteen mammalian AQPs (AQP0-AQP12) are known to be widely expressed in various epithelia and endothelia in many organs. AQPs form tetramers in membranes, each monomer of which contains six transmembrane α-helical domains, with the amino and carboxyl termini being located on the cytoplasmic surface of the membrane [1,2]. The narrow water-permeable pore in each monomer is formed by two highly conserved short hydrophobic portions of amino acid residues called asparagine-proline-alanine motifs [3]. Their primary function is to regulate water flow across the plasma membranes of cells.

We previously reported that AQP1 protein expression is enhanced in the tunica vaginalis of patients with adult-onset non-communicating hydrocele testis compared with controls [4] and may contribute to the development of non-communicating hydrocele testis. In other words, the baseline influx of fluid into the space between the tunica vaginalis and testis is increased by enhanced expression of a water channel protein, AQP1. Accordingly, it is assumed that as the absorption of solution in the tunica vaginalis by the lymphatic vessels decreases with aging, the amount of fluid storage within the tunica vaginalis increases, facilitating the formation of hydrocele testis. Tanriverdi et al. also reported increased expression of AQP1 protein in the tunica vaginalis in pediatric cases of non-communicating hydrocele testis [5], supporting this hypothesis.

However, the reason why AQP1 protein expression in the tunica vaginalis is enhanced in patients with non-communicating hydrocele testis has remained unclear. Thus, we performed genetic and epigenetic analyses of the *AQP1* gene in the tunica vaginalis of patients with adult-onset non-communicating hydrocele testis to elucidate the cause of enhanced AQP1 protein expression.

Specifically, the genotype was determined for Tag single-nucleotide polymorphisms (SNPs) representing the *AQP1* gene and SNPs in the 5’-upstream region of the *AQP1* gene. Then, by performing association analysis, the applicability of various genetic models was investigated for each SNP. Moreover, the methylation rate of 17 CpG sites was examined for the CpG island related to the *AQP1* gene.

An SNP is a variation of a single nucleotide in the genome sequence of an organism compared with the population, where the variation is found at a frequency of 1% or more within the population. The human genome contains approximately 3 billion base pairs, and SNPs occur in approximately one in every 1,000 base pairs. SNPs can be used as DNA markers to examine the genetic background, and SNP-based linkage and association analysis can help identify disease susceptibility genes. If two SNPs are in strong linkage disequilibrium, information about one SNP can provide information about the other. Thus, a representative SNP chosen to characterize a region of the genome is called a Tag SNP. Tag SNPs save cost and effort by eliminating the need to examine all SNPs in a region.

A CpG island is a region of the genome where CpG dinucleotides occur more frequently than in other regions of the genome. By definition, a CpG island is at least 200 base pairs long, has a GC content of at least 50%, and has an observed-to-expected CpG ratio >0.6 [6]. CpG islands are typically located at or near the transcription start site of 70% of human genes, particularly housekeeping genes [7]. The CpG sites in the CpG islands of promoters are unmethylated when the genes are expressed, and on the contrary, methylation of CpG sites in the promoter of a gene is supposed to inhibit gene expression.

CpG island shores are 2,000 base pair-long regions located on both sides of a CpG island, and gene expression levels are found to be negatively associated with methylation levels at CpG island shores [8]. Thus, this may be associated with tissue-specific gene expression.

## Materials and methods

Patients and samples

In this case-control study, 119 male patients (66 with hydrocele testis and 53 controls) were enrolled. Among them, 105 were from Kanto Rosai Hospital and 14 were from Toshiba Hospital. Their ages were 65.1 ± 14.5 and 66.1 ± 16.2 (p = 0.73, unpaired t-test) for hydrocele testis patients and controls, respectively. If yellowish hydrocele fluid was identified at the time of surgery, the diagnosis of hydrocele testis was determined regardless of its volume. The volume of hydrocele fluid measured was less than 10 mL in 20 cases, 10-100 mL in 15 cases, and more than 100 mL in 31 cases.

Genomic DNA was extracted from frozen (n = 86) and paraffin-embedded (n = 21) tunica vaginalis samples excised at surgery of hydrocele testis, other intrascrotal lesions, and bilateral orchiectomy for prostate cancer using a DNA Extractor® TIS Kit (296-67701, Wako, Osaka, Japan) and TaKaRa Dexpat™ Easy Kit (9104, TaKaRa). In addition, genomes were extracted from whole blood samples of 12 male patients with hydrocele testis using a DNA Extractor® WB-Rapid Kit (293-54803, Wako, Osaka, Japan).

Selection of Tag SNPs

Using the HapMap site [9] with a condition of r^2^ ≥ 0.8 and MAF ≥ 0.1, six Tag SNP sites for the human *AQP1* gene located on chromosome 7 were determined. These Tag SNP sites are listed in Table 1. The difference in minor allele frequency (MAF) between this study conducted on Japanese and 1000 Genomes, which is composed of multiple races, may be due to racial differences.

**Table 1:** SNPs investigated in the study. Chr: chromosome; MAF: minor allele frequency; SNP: single-nucleotide polymorphism

SNPs	Position on chr7 (GrCh38)	MAF (1000 Genomes)	MAF (present study)
SNPs in the 5’-upstream region			
rs28362689 (C>-)	30910928	0.021	0.076
rs1476597 (G>C)	30911127	0.399	0.429
rs2075574 (C>T)	30911129	0.361	0.348
Tag SNPs			
rs1004317 (A>G)	30917243	0.434	0.322
rs17159702 (T>C)	30919387	0.482	0.460
rs765840 (T>A)	30919869	0.303	0.378
rs11537660 (T>C)	30924154	0.337	0.278
rs1049305 (G>C)	30924207	0.448	0.361
rs11537661 (C>T)	30924530	0.170	0.185

Analysis of Tag SNPs by the Invader® assay

For 88 genomic DNAs (55 from frozen tunica vaginalis, 21 from paraffin-embedded tunica vaginalis, and 12 from whole blood), the genotypes of the Tag SNPs were determined by the Invader® assay [10,11] using a thermal cycler (GeneAmp PCR System 9700, Life Technologies, Carlsbad, CA) and Step One Plus Real-Time PCR System (Life Technologies, Carlsbad, CA).

Analysis of the 5’-upstream region of the *AQP1 *gene

For 92 genomic DNAs (82 from frozen tunica vaginalis and 10 from whole blood), the 5’-upstream region of the *AQP1* gene was amplified using a thermal cycler (Wako WK-0518, Wako, Osaka, Japan) and volumes of 50 μL with 0.3 μM of sense/antisense primers (5’-tcccagagactggaatgctgagcca-3’/5’-gcttcttcttgaactcgctggcca-3’), 1 unit of KOD Plus Ver.2 polymerase (Toyobo, Osaka, Japan), 4% dimethyl sulfoxide, and a buffer supplied with the enzyme as follows: 94°C for two minutes, 36 cycles (98°C for 10 seconds for denaturing, 68°C for two minutes for annealing and extension) of two-step polymerase chain reaction (PCR). Amplified PCR products (10 μL) were resolved by electrophoresis in 1% agarose gel. PCR products were directly Sanger sequenced with three sequencing primers, namely, 5’-cagggctcctcagaggaaagg-3’, 5’-gtggggctgccattccttccacc-3’, and 5’-tggcagggggcttggcctgagac-3’, and the genotypes of the SNPs were determined. The analyzed SNPs are listed in Table 1.

Analysis of genetic models

For each SNP in the *AQP1* gene and its 5’-upstream region, we analyzed the applicability of four different genetic models [12] by association analysis. In the recessive, dominant, and multiplicity models, minor alleles were assumed to be risk alleles for hydrocele testis, and in the over-dominant model, heterozygosity was assumed to be a risk genotype.

Analysis of CpG island methylation

Methylation of the CpG island of the *AQP1* gene was evaluated using genomes extracted from the tunica vaginalis. A 495 bp CpG island was detected in the *AQP1* gene and its upstream sequence using Methyl Primer Express v1.0 software. The island was mostly located within the coding region of exon1, downstream of the core promoter. Furthermore, the GC content was 60.6%, and the observed-to-expected CpG ratio was 59.4%. CpG islands were not identified within the promoter region of the *AQP1* gene. Sixty-six genomes (32 hydrocele testis and 34 controls) extracted from frozen tunica vaginalis were bisulfited using an EpiSight™ Bisulfite Conversion Kit Ver.2 (Fujifilm Wako Pure Chemicals, Osaka, Japan) and amplified with two pairs of bisulfite primers (5’-gaagaagtttttttggagggtag-3’/5’-tctaacaactaaacaacaaccccaatat-3’, 5’-ggggttgttgtttagttgttaga-3’/5’-cccaaaaacaccccactc-3’) using a PyroMark® PCR Kit (Qiagen Japan, Tokyo, Japan) at an annealing temperature of 56℃, following the manufacturers’ instructions. Pyrosequencing was performed on the amplified products, and the percentage of methylation was measured for 17 CpG sites using PyroMark Q24 (Qiagen, Hilden, Germany). The sequences analyzed after bisulfitization were yggyggtttaggataaygtgaaggtgtyg, tygttaygttggygtagagtgtgggttatattagyggyg, ttatygtttagtgygtgggggttatygtygttatyg, and tygtttggtygtaatgayg.

Statistical analysis

Statistical analysis was performed using R. In the genetic model for SNPs, Fisher’s exact test was used. Welch t-test was used for the methylation rate of each CpG site.

## Results

Genotyping of SNPs and analysis of genetic models

There was no significant association between each SNP and hydrocele testis for any of the genetic models (Table 2). Similar results were obtained when the volume of fluid separating hydrocele testis and control was set at 10, 50, and 100 mL (data not shown). Moreover, multivariate analysis using age as a covariate revealed no significant association between each SNP and hydrocele testis in either genetic model (data not shown).

**Table 2:** Genotyping of SNPs and analysis of genetic models. p-values obtained using Fisher’s exact test. OR: odds ratio; CI: confidence interval; Inf: infinite; M: major allele at the SNP site; m: minor allele at the SNP site; SNP: single-nucleotide polymorphism

SNPs	Condition	n	Genotype			Recessive model		Dominant model		Over-dominant model		Multiplicity model	
			MM	Mm	mm	OR (95% CI)	P-value	OR (95% CI)	P-value	OR (95% CI)	P-value	OR (95% CI)	P-value
rs28362689	Hydrocele	51	45	4	2	Inf	0.501	0.78	0.761	0.50	0.332	1.08	1.000
C>-	Control	41	35	6	0	(0.15–Inf)		(0.19–3.19)		(0.10–2.29)		(0.31–3.94)	
rs1476597	Hydrocele	51	20	20	11	0.98	1.000	0.72	0.516	0.75	0.530	1.08	1.000
G>C	Control	41	13	19	9	(0.32–3.03)		(0.27–1.86)		(0.30–1.86)		(0.31–3.94)	
rs2075574	Hydrocele	51	23	22	6	0.78	0.761	0.86	0.833	0.97	1.000	0.84	0.637
C>T	Control	41	17	18	6	(0.19–3.19)		(0.35–2.14)		(0.39–2.41)		(0.43–1.63)	
rs1004317	Hydrocele	54	24	25	5	1.02	1.000	1.04	1.000	1.03	1.000	1.07	0.869
A>G	Control	33	15	15	3	(0.18–7.04)		(0.40–2.71)		(0.40–2.71)		(0.53–2.19)	
rs17159702	Hydrocele	54	14	31	9	0.90	1.000	0.92	1.000	0.99	1.000	0.94	0.876
C>T	Control	33	8	19	6	(0.25–3.44)		(0.29–2.75)		(0.38–2.60)		(0.49–1.82)	
rs765840	Hydrocele	49	15	31	3	0.48	0.431	1.29	0.637	1.61	0.362	0.99	1.000
A>T	Control	33	12	17	4	(0.07–3.04)		(0.46–3.63)		(0.60–4.36)		(0.50–2.00)	
rs11537660	Hydrocele	54	28	22	4	2.61	0.645	0.83	0.827	0.69	0.509	0.99	1.000
T>C	Control	34	16	17	1	(0.24–133.80)		(0.32–2.12)		(0.27–1.78)		(0.48–2.08)	
rs1049305	Hydrocele	50	20	23	7	2.50	0.306	0.86	0.820	0.63	0.372	1.10	0.869
C>G	Control	33	12	19	2	(0.44–26.26)		(0.31–2.32)		(0.23–1.66)		(0.55–2.22)	
rs11537661	Hydrocele	53	35	16	2	Inf	0.529	0.94	1.000	0.79	0.636	1.01	1.000
C>T	Control	31	20	11	0	(0.11–Inf)		(0.34–2.66)		(0.28–2.27)		(0.42–2.56)	

Analysis of CpG island methylation

**Figure 1:**
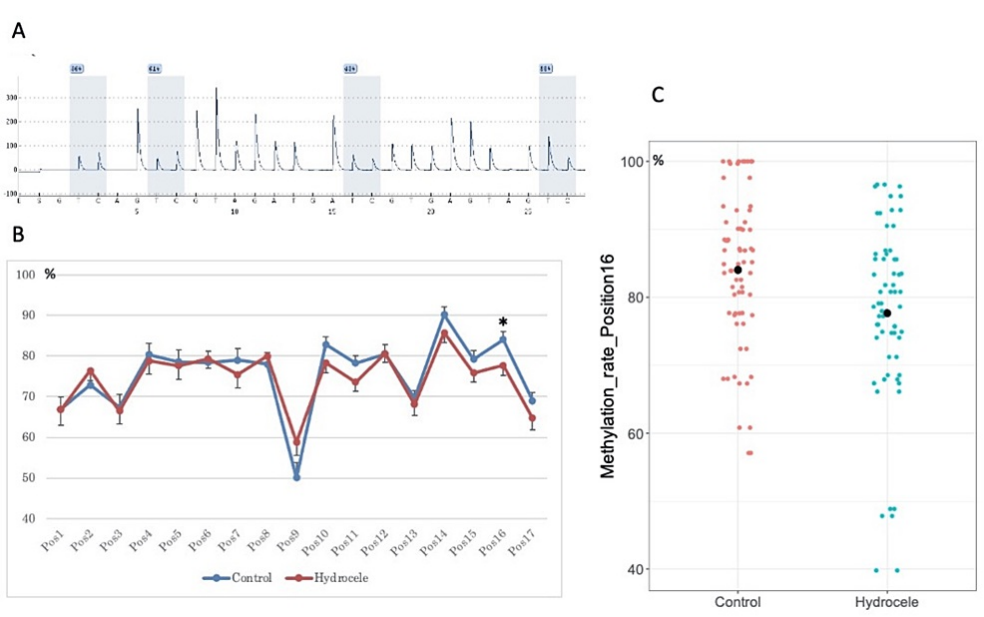
Methylation analysis of CpG sites. (A) Representative result of pyrosequencing used to measure methylation of CpG sites. Numbers: methylation rate. (B) Average methylation rates of each CpG site. Pos: position from 5’ to 3’, vertical axis: methylation rate, asterisk: p-value <0.05, bars show standard error. (C) A jitter plot showing the methylation rate at CpG position 16. Vertical axis: methylation rate, black circle: mean.

The average methylation rate of the 17 CpG sites evaluated was not significantly different between controls and hydrocele testis (75.6 ± 9.2% vs. 74.3 ± 7.9%, p = 0.561, Welch t-test). Considering the individual CpG sites, the methylation rate was lower in hydrocele testis than in controls at CpG position 16 (84.1 ± 11.3% vs. 77.6 ± 13.5%, p = 0.041, Welch t-test), but at other CpG sites, there was no significant difference in methylation rates between controls and hydrocele testis (Figures 1A-1C).

## Discussion

Most of the CpG island detected in this study was located within the coding region of exon1, downstream of the core promoter. Thus, although this was detected as a CpG island, it may be closer to a CpG island shore. CpG islands were not detected in the promoter region of the *AQP1* gene.

Overall, although there was no difference in the average methylation rate of the CpG sites within the CpG island between hydrocele testis and controls, one CpG site showed a decreased methylation rate in hydrocele testis. This hypomethylation may contribute to enhanced AQP1 protein expression in the tunica vaginalis of patients with hydrocele testis and the development of hydrocele testis. However, the methylation status of the CpG island shore, which extends further on both sides of the CpG island, should be investigated more extensively to clarify its relationship with enhanced AQP1 protein expression.

SNPs in the *AQP1* gene and its 5’-upstream region were not associated with the development of hydrocele testis in this study. Eight SNPs were examined herein, of which a few have been reported to be associated with diseases and pathological conditions. A minor allele of rs2075574, located in the 5’-upstream region of the *AQP1* gene, was associated with decreased peritoneal ultrafiltration and increased mortality in peritoneal dialysis patients, as well as decreased promoter activity of the *AQP1* gene and decreased AQP1 protein expression [13]. This suggests an association between the rs2075574 major allele and hydrocele testis, but this was not the case in the present study. Moreover, the major allele of rs17159702, located in the intron 1 of the *AQP1* gene, was previously associated with sudden infant death syndrome [14]. The major allele of rs1049305, located in the 3’-untranslated region of the *AQP1* gene, was reported to be a risk SNP for malignant mesothelioma [15], and the minor allele of rs1049305 was associated with water retention in cirrhotic patients [16] and running performance in marathons [17].

**Figure 2:**
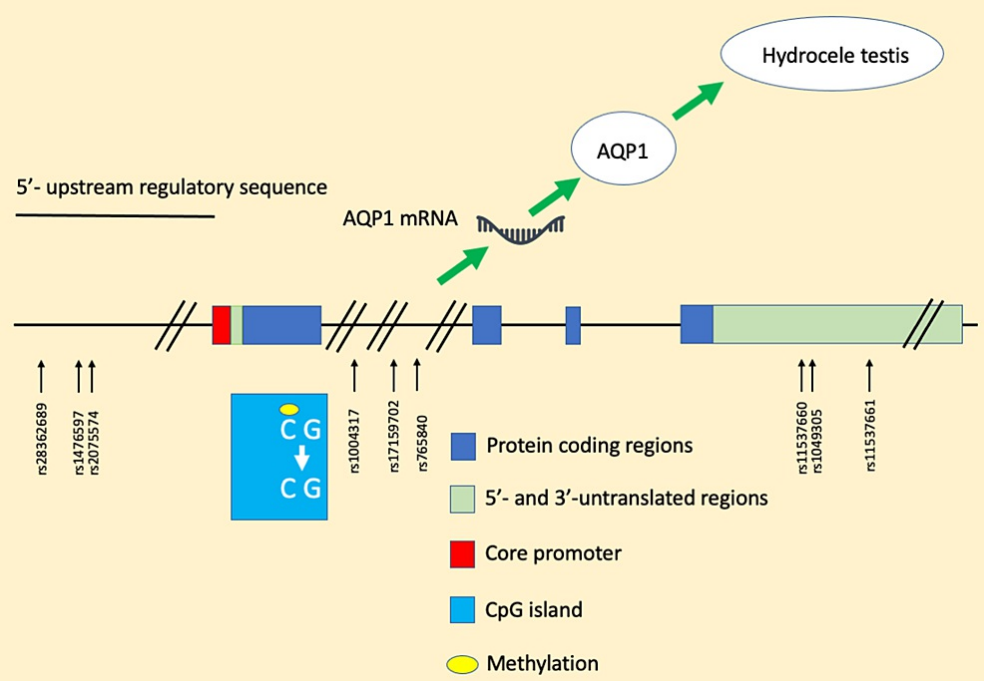
Graphical summary.

In the present study, the relationship between genetic and epigenetic changes in the *AQP1* gene and AQP1 protein production was not directly examined, but hydrocele testis was analyzed as a target factor. The development of hydrocele testis involves not only an increase in fluid influx due to an increase in AQP1 protein but also a factor of decreased fluid absorption due to changes in the lymphatic vessels of the tunica vaginalis. Thus, it cannot be said that SNPs associated with the *AQP1* gene do not cause changes in AQP1 protein levels. If fluid absorption from the lymphatic vessels is sufficient, even a small increase in AQP1 protein levels does not result in fluid retention as a subtraction. Figure 2 presents a graphical summary of the study findings.

There are a few limitations of this study. Decreased methylation of one CpG site in the CpG island was suggested as a contributing factor to the increase in AQP1 protein in the tunica vaginalis. Further pursuit will require detailed methylation analysis in the CpG shore, which extends on both sides of the CpG island.

## Conclusions

Adult-onset, primary, non-communicating hydrocele testis involves the accumulation of clear fluid between the tunica vaginalis and testis with the enhanced influx of fluid in the space. The enhanced influx can be contributed by the enhanced water channel AQP1 protein in the tunica vaginalis. There was a significant decrease in the methylation rate at one of the CpG sites in the CpG island associated with the *AQP1* gene in the tunica vaginalis of patients with non-communicating hydrocele testis. This may increase AQP1 protein expression and contribute to the formation of hydrocele testis. SNPs related to the *AQP1* gene were not associated with hydrocele testis.
